# Supplementary material for: Suffering from chronic tinnitus, chronic neck pain, or both: Does it impact the presence of signs and symptoms of central sensitization?
Source: PLoS One. 2023 Aug 24;18(8):e0290116. doi: 10.1371/journal.pone.0290116 (PMC10449148; doi:10.1371/journal.pone.0290116)
Supplement: S1 Table — (DOCX) [file pone.0290116.s001.docx]

***S1*: Standardization of test locations for pressure pain and heat pain thresholds**

| **Order** | **Measurement location** | **Standardization** |
| --- | --- | --- |
| 1 | Articular pillar of the C5–6 zygapophyseal joints | The patient was lying prone and a mark was placed 1 cm lateral to the mid distance between the spinous processes of C5 and C6, which were identified by palpation. |
| 2 | Masseter muscle | The patient was lying on the non-tested side. A mark was placed on the crossing of the line between the tragus of the ear and the corner of the mouth and the line between the corner of the mandibulae and the lateral corner of the eye. |
| 3 | Frontalis muscle | Patient was lying supine and a mark was placed on the forehead, 5 centimeters above the pupil of the eye. |
| 4 | Tibialis Anterior muscle | The patient was sitting with back support, the knee bended and the plantar surface of the foot resting on the treatment table. A mark was placed 2.5 cm lateral and 5 cm inferior to the tibial tubercle. Resisted active dorsiflexion confirmed correct muscle belly palpation. |
| 5 | Lateral elbow (proximal region of the muscle belly of extensor carpi radialis longus for the forearm) | The patient was sitting with back support and the arm supported in 90 ° elbow flexion and 90° shoulder abduction by the treatment table. A mark was placed 1 centimeter lateral and 1 centimeter caudal of the lateral epicondyle. |
