# Supplementary material for: Suffering from chronic tinnitus, chronic neck pain, or both: Does it impact the presence of signs and symptoms of central sensitization?
Source: PLoS One. 2023 Aug 24;18(8):e0290116. doi: 10.1371/journal.pone.0290116 (PMC10449148; doi:10.1371/journal.pone.0290116)
Supplement: S1 File — (PDF) [file pone.0290116.s002.pdf]

Document A
INTERVENTIONEEL ACADEMISCH ONDERZOEK

COMMISSIE VOOR MEDISCHE ETHIEK

telefoon
+32 (0)9 332 56 13 | +32 (0)9 332 33 36 | +32 (0)9 332 68 55

fax
+32 (0)9 332 49 62

e-mail
ethisch.comite@uzgent.be

VERZOEK TOT ADVIES VAN DE COMMISSIE VOOR MEDISCHE ETHIEK OVER EEN ONDERZOEKS-PROJECT BIJ DE MENS

EUDRACT NUMMER (indien interventioneel geneesmiddelenonderzoek): NVT

1. Titel van het onderzoek

Modulating mechanisms in patients with chronic subjective tinnitus and/or chronic pain

2. Gegevens van de onderzoeker(s)

[de eerste onderzoeker moet een persoon zijn die vast verbonden is aan de dienst (geen ASO) of universiteit]

Naam: Meeus Voornaam: Mira

Functie: ZAP

Faculteit/vakgroep: Revalidatiewetenschappen

Telefoon/gsm: 09 332 69 19

E-mail: mira.meeus@ugent.be

Naam UZ-diensthoofd of vakgroepvoorzitter: Prof. dr. Dirk Cambier

3. Gegevens van de medewerker(s) aan de studie

Naam: De Meulemeester Voornaam: Kayleigh

Functie: Doctorassistent

Faculteit/vakgroep: Revalidatiewetenschappen

Telefoon/gsm: 09 332 53 74

E-mail: kayleigh.demeulemeester@ugent.be

Naam UZ-diensthoofd of vakgroepvoorzitter: Prof. dr. Dirk Cambier

Naam: Lenoir Voornaam: Dorine

Functie: Doctoraatstudente

Faculteit/vakgroep: Revalidatiewetenschappen

Telefoon/gsm: 09 332 53 74

E-mail: dorine.lenoir@ugent.be

Naam UZ-diensthoofd of vakgroepvoorzitter: Prof. dr. Dirk Cambier

Naam: Cagnie Voornaam: Barbara

Functie: ZAP

Faculteit/vakgroep: Revalidatiewetenschappen

Telefoon/gsm: 09 332 52 65

E-mail: barbara.cagnie@ugent.be

Naam UZ-diensthoofd of vakgroepvoorzitter: Prof. dr. Dirk Cambier

Naam: Keppler Voornaam: Hannah

Functie: ZAP

Faculteit/vakgroep: Revalidatiewetenschappen

Telefoon/gsm: 09 332 04 08

E-mail: Hannah.keppler@ugent.be

Naam UZ-diensthoofd of vakgroepvoorzitter: Prof. dr. Dirk Cambier

Naam: Declerck Voornaam: Ann

Functie: Arts

Faculteit/vakgroep: Hoofd en Huid

Telefoon/gsm:

E-mail: Ann.Deklerck@UGent.be

Naam UZ-diensthoofd of vakgroepvoorzitter: Prof. Dr. Ingeborg Dhooge

Naam: Dhooge Voornaam: Ingeborg

Functie: Arts

Faculteit/vakgroep: Hoofd en Huid

Telefoon/gsm: 09 332 23 31

E-mail: Ingeborg.Dhooge@UGent.be

Naam UZ-diensthoofd of vakgroepvoorzitter: Prof. Dr. Ingeborg Dhooge

Naam: Degeest Voornaam: Sofie

Functie: Doctor-assistent

Faculteit/vakgroep: Revalidatiewetenschappen

Telefoon/gsm: 09 332 59 63

E-mail: Sofie.degeest@ugent.be

Naam UZ-diensthoofd of vakgroepvoorzitter: Prof. dr. Dirk Cambier

Naam: Kestens Voornaam: Katrien

Functie: Doctoraatsstudente

Faculteit/vakgroep: Revalidatiewetenschappen

Telefoon/gsm:

E-mail: Katrien.kestens@ugent.be

Naam UZ-diensthoofd of vakgroepvoorzitter: Prof. dr. Dirk Cambier

4. Soort onderzoek

☒ interventioneel onderzoek

- ☐ met geneesmiddel (alle items van toepassing aanduiden)
  - ☐ fase I
  - ☐ fase II

- ☐ fase III
- ☐ fase IV
- ☐ proef voor gentherapie en somatische celtherapie
- ☐ proef met geneesmiddelen die genetisch gewijzigde organismen bevatten
- ☐ proef met celtherapie met xenogenen

☒ andere

- ☐ medical device
- ☐ bloedafname, RX, …
- ☒ andere: klinisch onderzoek, quantitative sensory testing (pijnonderzoek door middel van drukpijndrempels, warmte detectie/pijndrempels, warmwaterbad), shear wave elastography (evaluatie stijfheid spierweefsel), Qualisys (3D bewegingsanalyse nek en kaak), cognitieve testen en luisterinspanningstest

5. Is het onderzoek

☒ diagnostisch	☐ therapeutisch
☒ fysiologisch	☒ fysiopathologisch
☐ morfologisch	☐ epidemiologisch

6. Is het onderzoek in België

☒ monocentrisch

☐ multicentrisch

☐ de Commissie voor Medische Ethiek UZ Gent is de centrale commissie

☐ ja (naam, adres, tel, fax en e-mail van andere Commissies voor Medische Ethiek die meewerken aan het onderzoek + naam van de lokale onderzoeker)
<Klik&TypTekst>

☐ neen (naam, adres, tel, fax en e-mail van de centrale Commissie voor Medische Ethiek)
<Klik&TypTekst>

7. Gaat dit onderzoek ook door in het buitenland

NVT

☐ in Europa – Welke zijn de deelnemende landen: <Klik&TypTekst>

☐ in de Verenigde Staten

8. Wordt deze studie financieel ondersteund?

☒ ja ☐ neen

☒ FWO/BOF

☐ farmaceutische industrie: <Klik&TypTekst>

☐ andere: <Klik&TypTekst>

9. Wie is de opdrachtgever van de studie die niet door de industrie wordt gesponsord?

☐ medewerker van het UZ Gent (naam en adres): <Klik&TypTekst>

☒ medewerker van de UGent (naam en adres): Prof. dr. Mira Meeus, Corneel Heymanslaan 10, 9000 Gent

☐ andere, specifieer (naam en adres): <Klik&TypTekst>

10. Geef een korte samenvatting van het protocol (minimum 30 zinnen/ een halve pagina en maximum één pagina), verstaanbaar voor mensen niet gespecialiseerd in de materie. Verwijs niet alleen naar een bijgevoegd protocol.

Tinnitus wordt gerapporteerd bij 10 tot 27 % van de bevolking, 30 % daarvan ervaart een negatieve invloed van tinnitus op hun dagelijks functioneren. De meest voorkomende vorm is **subjectieve tinnitus**, dit wordt gedefinieerd als een fantoomgeluid in de afwezigheid van een interne of externe geluidsbron. Momenteel wordt subjectieve tinnitus voornamelijk behandeld door psychotherapie, medicatie, maskeerapparaten en cognitieve gedragstherapie maar effectiviteitsstudies tonen slechts een **klein tot matig positief effect** aan. Een oorzaak van dit beperkt therapie-effect kan verklaard worden door het feit dat tinnitus een **klinisch heterogeen en multifactorieel probleem** is dat beïnvloed/gemoduleerd kan worden door disfuncties op verschillende fysiologische en psychologische niveaus dewelke vergelijkbaar zijn met de mechanismen die een belangrijke invloed hebben op **chronische pijn**. De meest besproken beïnvloedende factoren hierbij zijn **musculoskeletale disfuncties** (problemen ter hoogte van de gewrichten en/of spieren in de nek-, hoofd- en kaakregio), symptomen indicatief voor **centrale sensitisatie** (verhoogde prikkelbaarheid van het centraal zenuwstelsel) en **cognitieve/psychosociale factoren** zoals stress, negatieve gedachten, slapeloosheid en verschillende persoonlijkheidsfactoren. Daarnaast werd ook reeds aangetoond dat geen verband bestaat tussen tinnituskarakteristieken (zoals intensiteit en toonhoogte), en de **tinnitus distress** die patiënten ervaren, wat doet vermoeden dat andere factoren bijdragen tot de mate van tinnitus distress.

De meeste studies over modulerende factoren bij tinnitus zijn meestal gebaseerd op **klinische evaluaties** en er zijn **geen studies** waarin de aanwezigheid van centrale sensitisatie onderzocht werd. Er is ook weinig kennis over de **mate** waarin deze modulerende factoren de tinnituskarakteristieken effectief beïnvloeden en hoe deze factoren onderling elkaar beïnvloeden.

Het **doel** van dit onderzoek is daarom om

1) De **relatie** tussen tinnitus distress en verschillende **modulerende factoren** na te gaan. Op deze manier kunnen de meest relevante modulerende factoren geïdentificeerd worden.

2) Objectieve en klinische maten voor modulerende factoren te gaan **vergelijken** tussen patiënten met **chronische subjectieve tinnitus en chronische musculoskeletale nek, hoofd- en/of kaakpijn**, patiënten met **chronische subjectieve tinnitus** alleen, patiënten met **chronische musculoskeletale nekpijn** alleen, alsook met **gezonde controlepersonen**. Op deze manier kan geëvalueerd worden of deze modulerende factoren geassocieerd zijn met tinnitus, pijn of beide.

3) Evaluatie van **objectieve metingen**, alsook hun **relatie met klinische metingen** van symptomen die representatief zijn voor de modulerende mechanismen bij patiënten met chronische subjectieve tinnitus. Op deze manier kan een klinische testbatterij opgesteld worden die kan gebruikt worden in de praktijk.

Om deze doelen te bereiken zal een **cross-sectioneel onderzoek** uitgevoerd worden waarin verschillende **uitkomstmaten** gemeten zullen worden in de 4 bovengenoemde groepen:

1) Tinnituskarakteristieken: hierbij zal de intensiteit, locatie en toonhoogte van de tinnitus geëvalueerd worden door een ervaren audioloog. Daarnaast zullen ook de **Tinnitus Functional Index** (hinder die patiënten ervaren in hun dagelijks functioneren), de **Tinnitus Sample Case History Questionnaire** (tinnituskenmerken en beïnvloedende factoren) en de **Hyperacusis Questionnaire** (overgevoeligheid voor geluid) afgenomen worden.

2) Musculoskeletale factoren: als klinische uitkomstmaten zal een **kinesitherapeutisch onderzoek** uitgevoerd worden bestaande uit de evaluatie van de mobiliteit van de nek- en kaakregio alsook palpatie van de nek-, kaak-, en hoofdmusculatuur. Als objectieve uitkomstmaten zullen de mobiliteit van de nek- en kaakregio gemeten worden via een **3D kinematische analyse** (Qualisys), alsook de stijfheid van de musculatuur met behulp van **shear wave elastography** (echografietechniek).

3) **Centrale sensitisatie:** als klinische uitkomstmaat zal de **Central Sensitization Inventory** afgenomen worden, dit is een vragenlijst die peilt naar zelf gerapporteerde klinische symptomen, indicatief voor centrale sensitisatie. Als objectieve uitkomstmaten zullen **drukpijndrempels, warmtedetectiedrempels, warmtepijndrempels** alsook **temporele summatie** (pijnrespons op herhaaldelijke stimuli) en **conditioned pain modulation** (pijninhibtie) gemeten worden.

4) **Cognitieve, gedrags- en pyschosociale factoren:** als klinische uitkomstmaten worden verschillende vragenlijsten afgenomen, namelijk: de **Short Form Health Survey-36 items** (levenskwaliteit), de **BIG 5 index 2** (persoonlijkheidskenmerken), de **Beck Depression Inventory** (mate van depressieve gevoelens), **Depression Anxiety and Stress Scale** (evaluatie van depressie, angst en stress), **Pain Catastrophizing Scale** (pijncognities), **Insomnia Severity Index** (mate van slapeloosheid), **Pittsburgh Sleep Quality Index** (slaapkwaliteit). Er worden **cognitieve testen** afgenomen die peilen naar het werkgeheugen (nazeggen van cijfers en letters, subtest WAIS-IV-NL), verwerkingssnelheid (tijd nodig om cijfers en letters na te zeggen), selectieve aandacht (letters detecteren, COTESS) en executieve functies (auditieve Stroop test). Ook een gedragsmatige luisterinspanningstest wordt afgenomen, en evalueert de cognitieve processen nodig om spraak te verstaan. De deelnemers zullen ook gevraagd worden om gedurende een week een **activity tracker** te dragen die kosteloos ter beschikking wordt gesteld, deze zal de slaapkwantiteit en kwaliteit alsook de mate van fysieke activiteit meten.

5) **Hersenactiviteit obv electro-encefalografie (EEG)**: Bij aanvang van het testmoment zal de **hersenactiviteit in rust** nagegaan worden (5 minuten resting-state EEG), alsook zal er EEG afgenomen worden **tijdens de evaluatie van temporele summatie en conditioned pain modulation**. Op basis van de resting-state EEG zal een **spectraal analyse** uitgevoerd worden, die vergeleken zal worden tussen de verschillende groepen. De metingen tijdens temporele summatie en conditioned pain modulation zullen gebruikt worden om **event-gerelateerde potentialen** mee te berekenen (die kennis verstrekken over de latentie en amplitudo van de hersenrespons op een prikkel) en opnieuw te vergelijken tussen de 4 groepen. Voor deze metingen zal een ANTneuro EEG systeem gebruikt worden, gekoppeld aan een EEG cap met 32 kanalen, geplaatst volgens het 10-20 systeem.

11. Wat zijn de argumenten (theoretische, experimentele of andere) die een voordeel laten verwachten van de te testen nieuwe methode, preparaat, … boven de bekende en reeds gebruikte?

Chronische subjectieve tinnitus is een frequent voorkomend probleem met een hoge impact in het dagelijks functioneren en levenskwaliteit in een subgroep van patiënten. De huidige behandelaanpak is beperkt in therapeutische effectiviteit, waarschijnlijk door een gebrek aan differentiatie en gericht aanpakken van de modulerende mechanismen binnen deze klinisch heterogene patiëntengroep. Identificatie van de meest relevante modulerende factoren zal het mogelijk maken om in de toekomst studies uit te voeren waarin het behandeleffect bij het aanpakken van de meest relevante factoren kan onderzocht worden. Momenteel is het fundamenteel onderzoek van de modulerende mechanismen bij subjectieve tinnitus beperkt en is er ook weinig kennis over de mate waarin deze mechanismen tinnituskarakteristieken beïnvloeden en hoe deze mechanismen elkaar beïnvloeden. Daarnaast kan, door het toetsen van klinische testen aan objectieve metingen, een betrouwbare klinische testbatterij uitgewerkt worden die in de klinische praktijk kan gebruikt worden en kunnen al dan niet parallelle mechanismen met de chronische pijnpopulatie geïdentificeerd worden. Op deze manier wordt het onderzoek en de behandeling bij patiënten met chronische tinnitus en/of chronische pijn geoptimaliseerd, wat kan leiden tot een afname in de impact op het dagelijks functioneren en levenskwaliteit van deze patiëntenpopulatie.

12. Werd een analoog onderzoek al elders uitgevoerd, in zijn geheel of gedeeltelijk?

Zo ja, waar? Wat was het resultaat? Waarom wordt het in deze studie hernomen?

In voorgaande onderzoeken werd reeds aangetoond dat tinnituskarakteristieken zoals luidheid, locatie en toonhoogte kunnen beïnvloed worden door musculoskeletale factoren zoals myofasciale triggerpunten in verschillende nek- en kaakspieren alsook door een beperkte mobiliteit in de nek- en kaakregio. Deze dysfuncties werden echter enkel op een klinische manier geëvalueerd zonder het uitvoeren van objectieve metingen.

- Michiels S, De Hertogh W, Truijen S, Van de Heyning P. Cervical spine dysfunctions in patients with chronic subjective tinnitus. Otol Neurotol Off Publ Am Otol Soc Am Neurotol Soc [and] Eur Acad Otol Neurotol. 2015 Apr;36(4):741–5.
- Michiels S, Van de Heyning P, Truijen S, Hallemans A, De Hertogh W. Prognostic indicators for decrease in tinnitus severity after cervical physical therapy in patients with cervicogenic somatic tinnitus. Musculoskelet Sci Pract. 2017 Jun 1;29:33–7.
- Sanchez TG, Rocha CB. Diagnosis and management of somatosensory tinnitus: review article. Clinics (Sao Paulo). 2011;66(6):1089–94.

Daarnaast hebben verschillende studies ook reeds een verband aangetoond tussen tinnitusluidheid en de impact op het dagelijks functioneren enerzijds en stress, depressie, persoonlijkheidskenmerken en slapeloosheid anderzijds.

- Gomaa MAM, Elmagd MHA, Elbadry MM, Kader RMA. Depression, Anxiety and Stress Scale in patients with tinnitus and hearing loss. Eur Arch Otorhinolaryngol. 2014 Aug;271(8):2177–84.
- Fioretti AB, Fusetti M, Eibenstein A. Association between sleep disorders, hyperacusis and tinnitus: evaluation with tinnitus questionnaires. Noise Health. 2013;15(63):91–5.
- Durai M, Searchfield G. Anxiety and depression, personality traits relevant to tinnitus: A scoping review. Vol. 55, International Journal of Audiology. Taylor and Francis Ltd; 2016. p. 605–15.

13. Zal een chemische substantie toegediend worden?

☐ ja ☒ neen

Zo ja:

a. Langs welke weg? <Klik&TypTekst>

b. Naam en oorsprong van de substantie: <Klik&TypTekst>

c. Aan wie wordt de receptie, opslag, verdeling en terugsturen van niet-gebruikte chemische substanties toevertrouwd? <Klik&TypTekst>

d. Zullen radio-isotopen toegediend worden?

☐ ja ☐ neen

Welke? <Klik&TypTekst>

14. Indien het om een nieuwe substantie gaat: heeft de onderzoeker kennisgenomen van het volledige toxicologische, dierfarmacologische en humane dossier?

☐ ja ☐ neen

NVT

15. Keuze van de proefpersonen:

a. Gezonden?

☒ ja ☒ neen

Patiënten lijdend aan: Chronische tinnitus en/of chronische nek, hoofd en/of kaakpijn

b. Zwangere vrouwen of vrouwen die tijdens het onderzoek zwanger kunnen worden?

☐ ja ☒ neen

c. Aantal proefpersonen in het UZ Gent: 212 (53 tinnituspatiënten met chronische pijn, 53 tinnituspatiënten zonder chronische pijn, 53 nekpijnpatiënten zonder tinnitus en 53 gezonde controles)

d. Aantal proefpersonen extern (in België): <Klik&TypTekst>

Opgelet: het experiment is enkel verzekerd voor het aantal dat hier opgegeven wordt.

Indien men extra deelnemers wil includeren, zal men dat via een amendement moeten aanvragen.

e. Leeftijd: 18-65

f. Geslacht: Man/vrouw

g. Hoe worden ze gerekruteerd? Via dienst audiologie UZ Gent + via flyers bij artsen, apothekers, kinesitherapeuten en social media

16. Wanneer verwacht men voordeel voor de deelnemer?

a. Heeft het experiment een diagnostisch of therapeutisch doel dat onmiddellijk voordeel voor de onderzochte zal brengen?

☐ ja ☒ neen

b. Maakt het experiment deel uit van een diagnostisch en therapeutisch plan waarvan men mag verwachten dat de resultaten binnen afzienbare tijd voor andere zieken nuttig zullen zijn?

☒ ja ☐ neen

c. Maakt het experiment deel uit van een geheel van onderzoeken waarvan het diagnostische of therapeutische belang niet onmiddellijk duidelijk is, maar er mag worden verwacht dat de resultaten later tot diagnostische of therapeutische toepassingen of tot een betere kennis van de fysiopathologische mechanismen zullen leiden?

☒ ja ☐ neen

17. Welke interventies zijn specifiek voor de studie (naast de standaardbehandelingen), hoe frequent en gedurende welke tijd?

a. Zuiver klinische evaluaties: klinisch kinesitherapeutisch onderzoek van de nek en kaakregio (eenmalig)

b. Functietests of dynamische proeven (eenmalig):

- Audiogram en psychoakoestische metingen ikv tinnitus en hyperacusis (dienst NKO/audiologie)
- Cognitieve testen
- Luisterinspanningstest
- 3D kinematische analyse (Qualisys Motion Capture System)
- Shear Wave Elastography (SuperSonic Aixplorer MACH 30)
- Drukpijndrempels (digitale algometer)
- Warmtedetectiedrempels (CHEPS toestel)
- Temporele summatie (CHEPS toestel)
- Conditioned pain modulation (CHEPS toestel + warmwaterbad (Versacool))
- Electro-encefalografie (ANTneuro EEG toestel)

c. Radiografische en/of isotopische investigaties: NVT

d. Bloedafnamen: NVT

e. Weefselafname: NVT

f. Andere: Vragenlijsten (eenmalig):

- Tinnitus Functional Index
- Tinnitus Sample Case History Questionnaire
- Hyperacusis Questionnaire
- Central Sensitization Inventory
- Short Form Health Survey-36 items
- BIG 5 index 2
- Beck Depression Inventory

- Depression Anxiety and Stress Scale
- Pain Catastrophizing Scale
- Insomnia Severity Index
- Pittsburgh Sleep Quality Index

18. Rekening houdend met de huidige gegevens van de wetenschap

a. Meent u dat deze studie:

☒ waarschijnlijk geen enkel risico inhoudt

☐ een mogelijk risico inhoudt.

Welk risico en de frequentie:

<Klik&TypTekst>

☐ zeer waarschijnlijk een risico inhoudt.

Welk risico en de frequentie:

<Klik&TypTekst>

b. Welke zijn de meest voorkomende bijwerkingen van het preparaat onder studie?
(de bijwerkingen moeten ook duidelijk vermeld worden in het informatie- en toestemmingsformulier van de deelnemer)

NVT

19. Informatie en toestemming van de proefpersonen

a. Wilsbekwame volwassenen

☒ ja ☐ neen

Wordt de toestemming van de proefpersonen verkregen na een klare en objectieve uiteenzetting van het doel van het onderzoek?

Schriftelijk:

☒ ja ☐ neen

Mondeling:

☒ ja ☐ neen

Zo neen, waarom niet?

<Klik&TypTekst>

Wordt in dat laatste geval de toestemming gegeven door anderen dan de proefpersonen?

☐ ja ☐ neen

Zo ja, door wie?

<Klik&TypTekst>

Zijn er speciale groepen: eigen studenten, eigen personeel?

Deze worden niet specifiek gerekruteerd maar bij toeval kan een student of personeelslid deelnemen aan de studie indien deze persoon voldoet aan de voorwaarden voor het onderzoek.

b. Wilsonbekwame volwassenen (= sommige psychiatrische patiënten, personen in de onmogelijkheid hun wil te uiten, …)

☐ ja ☒ neen

Wordt de toestemming gegeven door anderen dan de proefpersonen?

☐ ja ☐ neen

Zo ja, door wie?

<Klik&TypTekst>

c. Kinderen

☐ ja ☒ neen

Wordt de toestemming gevraagd van hun wettelijke verantwoordelijken?

☐ ja ☐ neen

Is er een informatie- en toestemmingsformulier voor kinderen vanaf 12 jaar voorzien?

☐ ja ☐ neen

20. Is het informatieformulier voor de proefpersonen in de bijlage gevoegd

☒ ja ☐ neen

Zo neen, waarom niet?

<Klik&TypTekst>

21. Is het formulier voor schriftelijke toestemming in de bijlage gevoegd?

☒ ja ☐ neen

Zo neen, waarom niet?

<Klik&TypTekst>

22. Zullen de personen in de loop van deze studie voortdurend onder medisch toezicht staan?

☐ ja ☒ neen

a. Wie is de toezichthoudende arts?

<Klik&TypTekst>

b. Zal dat toezicht, zo nodig, verzekerd kunnen worden tijdens de uren die op de studie volgen?

☐ ja ☐ neen

c. Als de persoon naar huis terugkeert tijdens de uren die op het onderzoek volgen, zal in geval van nood snel contact met een arts kunnen opgenomen worden?

☐ ja ☐ neen

d. Naam van die arts?

<Klik&TypTekst>

23. Is er voor het onderzoek een verzekering afgesloten conform de Belgische wet van 7/5/2004?

(het verzekeringscertificaat moet bij de aanvraag gevoegd worden indien niet verzekerd door UZ Gent/UGent)

☒ ja

Door welke verzekeringspolis bent u verzekerd? (verwijzen naar een bijgevoegd document volstaat niet)

☐ UZ Gent

☒ UGent

☐ andere + omvang van de dekking: <Klik&TypTekst>

☐ neen, waarom niet? <Klik&TypTekst>

24. Einddatum experiment

Datum: woensdag 31 december 2025

Let wel: elk experiment op mensen na de einddatum is niet meer gedekt door de verzekering zodat op dat ogenblik u in overtreding bent met de wettelijke beschikkingen.
U kan het experiment wel verlenging mits een nieuwe aanvraag.

25. Financiële overeenkomst

Indien een definitieve financiële overeenkomst nog niet beschikbaar is, kan een budgetvoorstel dat tegen-getekend is door een vertegenwoordiger van de financierder + onderzoeker volstaan). Indien het bedrag van de definitieve financiële overeenkomst hoger is dan het ingediende budgetvoorstel, moet die definitieve financiële overeenkomst alsnog ter goedkeuring voorgelegd worden aan de Commissie voor Medische Ethiek.

☒ niet van toepassing

☐ aanwezig met volgende onderverdeling:

- ☐ ereloon: <Klik&TypTekst>
- ☐ vergoeding voor technische prestaties: <Klik&TypTekst>
