# Supplementary material for: Suffering from chronic tinnitus, chronic neck pain, or both: Does it impact the presence of signs and symptoms of central sensitization?
Source: PLoS One. 2023 Aug 24;18(8):e0290116. doi: 10.1371/journal.pone.0290116 (PMC10449148; doi:10.1371/journal.pone.0290116)
Supplement: S2 File — (DOCX) [file pone.0290116.s003.docx]

| Document AINTERVENTIONAL ACADEMIC RESEARCH | | | |
| --- | --- | --- | --- |
|  | | | |
|  | | | |
| **COMMISSION FOR MEDICAL ETHICS** | | | |
| **telephone**  +32 (0)9 332 56 13 \| +32 (0)9 332 33 36 \| +32 (0)9 332 68 55 | | **fax**  +32 (0)9 332 49 62 | **e-mail**  ethisch.comite@uzgent.be |
|  |  | | |

**REQUEST FOR MEDICAL ETHICS COMMITTEE OPINION ON A HUMAN RESEARCH PROJECT**

**EUDRACT NUMBER** (if interventional drug research): not applicable

1. Title of the study

Modulating mechanisms in patients with chronic subjective tinnitus and/or chronic pain

**2. Details of the investigator(s). [the initial investigator must be a person permanently attached to the department (not an ASO) or university].**

Name: Meeus First name: Mira

Function: ZAP

Faculty/department: Rehabilitation Sciences

Telephone: 09 332 69 19

E-mail: mira.meeus@ugent.be

Name UZ department head or department chairperson: Prof. dr. Dirk Cambier

**Details of contributor(s) to the study.**

Name: De Meulemeester First name: Kayleigh

Function: Doctor assistent

Faculty/department: Rehabilitation Sciences

Telephone: 09 332 53 74

E-mail: kayleigh.demeulemeester@ugent.be

Name UZ department head or department chairperson: Prof. dr. Dirk Cambier

Name: Lenoir First name: Dorine

Function: PhD student

Faculty/department: Rehabilitation Sciences

Telephone: 09 332 53 74

E-mail: dorine.lenoir@ugent.be

Name UZ department head or department chairperson: Prof. dr. Dirk Cambier

Name: Cagnie First name: Barbara

Function: Full-time professor

Faculty/department: Rehabilitation Sciences

Telephone: 09 332 52 65

E-mail: barbara.cagnie@ugent.be

Name UZ department head or department chairperson: Prof. dr. Dirk Cambier

Name: Keppler First name: Hannah

Function: Full-time professor

Faculty/department: Rehabilitation Sciences

Telephone: 09 332 04 08

E-mail: Hannah.keppler@ugent.be

Name UZ department head or department chairperson: Prof. dr. Dirk Cambier

Name: Declerck First name: Ann

Function: Medical doctor

Faculty/department: Head and skin

E-mail: [Ann.Deklerck@UGent.be](mailto:Ann.Deklerck@UGent.be)

Name UZ department head or department chairperson: Prof. Dr. Ingeborg Dhooge

Name: Dhooge First name: Ingeborg

Function: Medical doctor

Faculty/department: Head and skin

Telehone: 09 332 23 31

E-mail: Ingeborg.Dhooge@UGent.be

Name UZ department head or department chairperson: Prof. Dr. Ingeborg Dhooge

Name: Degeest First name: Sofie

Function: Doctor-assistent

Faculty/department: Rehabilitation Sciences

Telephone: 09 332 59 63

E-mail: Sofie.degeest@ugent.be

Name UZ department head or department chairperson: Prof. dr. Dirk Cambier

Name: Kestens First name: Katrien

Function: PhD student

Faculty/department: Rehabilitation Sciences

E-mail: Katrien.kestens@ugent.be

Name UZ department head or department chairperson: Prof. dr. Dirk Cambier

1. Type of research

interventionel research

with medication (mark all items that apply)

phase I

phase II

phase III

phase IV

trial for gene therapy and somatic cell therapy  trial of drugs containing genetically modified organisms

cell therapy trial with xenogens

others

medical device

blood sampling, RX, …

others: clinical examination, quantitative sensory testing (pain assessment using pressure pain thresholds, heat detection/pain thresholds, hot water bath), shear wave elastography (muscle tissue stiffness evaluation), Qualisys (3D motion analysis neck and jaw), cognitive testing and listening effort test

1. Is the research project:

diagnostic  therapeutic

fysiologic  physiopathologic

morphologic  epidemiologic

1. Is the research project in Belgium:

monocentric

multicentric

the Committee for Medical Ethics UZ Gent is the central committee  Yes (name, address, tel, fax and e-mail of other Medical Ethics Committees that are

collaborate in the study + name of local investigator)

no (name, address, tel, fax and e-mail of the Central Committee on Medical Ethics

1. Does this research continue abroad?

Not applicable

☐ in Europe - Which are the participating countries:<Click&TypeText>

☐ in the United States

1. Is this study financially supported?

yes  no

FWO/BOF

pharmaceutical industry:

others:

1. Who is the sponsor of the study not sponsored by industry?

☐ UZ Gent employee (name and address): <Click&TypText>

☒ UGent staff member (name and address): Prof. Dr. Mira Meeus, Corneel Heymanslaan 10, 9000 Ghent

☐ other, please specify (name and address):

1. Provide a brief summary of the protocol (minimum 30 sentences/half a page and maximum one page), understandable to people not specialized in the subject matter. Do not simply refer to an attached protocol.

Tinnitus is reported in 10-27% of the population, 30% of whom experience a negative impact of tinnitus on their daily functioning. The most common form is subjective tinnitus, which is defined as a phantom sound in the absence of an internal or external sound source. Currently, subjective tinnitus is mainly treated by psychotherapy, medication, masking devices and cognitive behavioral therapy but effectiveness studies show only a small to moderate positive effect. One cause of this limited therapy effect can be explained by the fact that tinnitus is a clinically heterogeneous and multifactorial problem that can be influenced/modulated by dysfunctions at different physiological and psychological levels which are similar to the mechanisms that have a significant impact on chronic pain. The most discussed influencing factors here are musculoskeletal dysfunctions (problems at the level of the joints and/or muscles in the neck, head and jaw region), symptoms indicative of central sensitization (increased excitability of the central nervous system) and cognitive/psychosocial factors such as stress, negative thoughts, insomnia and various personality factors. In addition, it has also already been shown that there is no relationship between tinnitus characteristics (such as intensity and pitch), and the tinnitus distress experienced by patients, suggesting that other factors contribute to the degree of tinnitus distress.

Most studies on modulating factors in tinnitus tend to be based on clinical evaluations, and there are no studies that have examined the presence of central sensitization. There is also little knowledge about the extent to which these modulatory factors effectively influence tinnitus characteristics and how these factors interact.

Therefore, the purpose of this study is to

1) Examine the relationship between tinnitus distress and various modulatory factors. In this way, the most relevant modulatory factors can be identified.

2) To compare objective and clinical measures of modulatory factors between patients with chronic subjective tinnitus and chronic musculoskeletal neck, head and/or jaw pain, patients with chronic subjective tinnitus alone, patients with chronic musculoskeletal neck pain alone, as well as with healthy control subjects. In this way, it can be evaluated whether these modulatory factors are associated with tinnitus, pain or both.

3) Evaluation of objective measurements, as well as their relationship with clinical measurements of symptoms representative of modulatory mechanisms in patients with chronic subjective tinnitus. In this way, a clinical test battery can be established that can be used in practice.

To achieve these goals, a cross-sectional study will be conducted in which several outcome measures will be measured in the 4 groups mentioned above:

1) Tinnitus characteristics: here the intensity, location and pitch of the tinnitus will be evaluated by an experienced audiologist. In addition, the Tinnitus Functional Index (annoyance experienced by patients in their daily functioning), the Tinnitus Sample Case History Questionnaire (tinnitus characteristics and influencing factors) and the Hyperacusis Questionnaire (hypersensitivity to sound) will also be taken.

2) Musculoskeletal factors: as clinical outcome measures, a physical therapy examination will be performed consisting of the evaluation of the mobility of the neck and jaw region as well as palpation of the neck, jaw, and head muscles. As objective outcome measures, the mobility of the neck and jaw region will be measured via 3D kinematic analysis (Qualisys), as well as the stiffness of the musculature using shear wave elastography (ultrasound technique).

3) Cognitive, behavioral and pyschosocial factors: several questionnaires are administered as clinical outcome measures, namely: the Short Form Health Survey-36 items (quality of life), the BIG 5 index 2 (personality traits), the Beck Depression Inventory (degree of depressive feelings), Depression Anxiety and Stress Scale (evaluation of depression, anxiety and stress), Pain Catastrophizing Scale (pain cognitions), Insomnia Severity Index (degree of insomnia), Pittsburgh Sleep Quality Index (sleep quality).

4) Cognitive tests are administered that gauge working memory (reciting numbers and letters, WAIS-IV-NL subtest), processing speed (time needed to recite numbers and letters), selective attention (letter detection, COTESS) and executive functions (auditory Stroop test). A behavioral listening effort test will also be administered, evaluating the cognitive processes required to understand speech. Participants will also be asked to wear an activity tracker provided free of charge for one week, it will measure sleep quantity and quality as well as the level of physical activity.

5) Brain activity by means of electroencephalography (EEG): At the beginning of the test moment, the brain activity at rest will be checked (5 minutes resting-state EEG), as well as EEG will be taken during the evaluation of temporal summation and conditioned pain modulation. On the basis of the resting-state EEG a spectral analysis will be performed, which will be compared between the different groups. The measurements during temporal summation and conditioned pain modulation will be used to calculate event-related potentials (which provide knowledge about the latency and amplitude of the brain response to a stimulus) and again compared between the 4 groups. An ANTneuro EEG system will be used for these measurements, coupled to an EEG cap with 32 channels, placed according to the 10-20 system.

1. What are the arguments (theoretical, experimental or other) that make one expect an advantage of the new method, preparation, ... to be tested over the known and already used ones?

Chronic subjective tinnitus is a frequent problem with a high impact in daily functioning and quality of life in a subgroup of patients. Current treatment approaches are limited in therapeutic effectiveness, probably due to a lack of differentiation and targeting of modulatory mechanisms within this clinically heterogeneous patient group. Identification of the most relevant modulatory factors will allow future studies in which the treatment effect in addressing the most relevant factors can be investigated. Currently, basic research of modulatory mechanisms in subjective tinnitus is limited and there is also little knowledge about the extent to which these mechanisms influence tinnitus characteristics and how these mechanisms influence each other. In addition, by testing clinical tests against objective measurements, a reliable clinical test battery can be developed that can be used in clinical practice and mechanisms, parallel or not, with the chronic pain population can be identified. In this way, research and treatment in patients with chronic tinnitus and/or chronic pain will be optimized, which may lead to a decrease in the impact on daily functioning and quality of life for this patient population.

1. Has analog research already been conducted elsewhere, in whole or in part? If so, where? What was the result? Why is it restated in this study?

Previous studies have already shown that tinnitus characteristics such as loudness, location and pitch can be influenced by musculoskeletal factors such as myofascial trigger points in various neck and jaw muscles as well as by limited mobility in the neck and jaw region. However, these dysfunctions were evaluated only in a clinical manner without performing objective measurements.

- Michiels S, De Hertogh W, Truijen S, Van de Heyning P. Cervical spine dysfunctions in patients with chronic subjective tinnitus. Otol Neurotol Off Publ Am Otol Soc Am Neurotol Soc [and] Eur Acad Otol Neurotol. 2015 Apr;36(4):741–5.
- Michiels S, Van de Heyning P, Truijen S, Hallemans A, De Hertogh W. Prognostic indicators for decrease in tinnitus severity after cervical physical therapy in patients with cervicogenic somatic tinnitus. Musculoskelet Sci Pract. 2017 Jun 1;29:33–7.
- Sanchez TG, Rocha CB. Diagnosis and management of somatosensory tinnitus: review article. Clinics (Sao Paulo). 2011;66(6):1089–94.

In addition, several studies have also already shown a link between tinnitus loudness and its impact on daily functioning on the one hand and stress, depression, personality traits and insomnia on the other.

- Gomaa MAM, Elmagd MHA, Elbadry MM, Kader RMA. Depression, Anxiety and Stress Scale in patients with tinnitus and hearing loss. Eur Arch Otorhinolaryngol. 2014 Aug;271(8):2177–84.
- Fioretti AB, Fusetti M, Eibenstein A. Association between sleep disorders, hyperacusis and tinnitus: evaluation with tinnitus questionnaires. Noise Health. 2013;15(63):91–5.
- Durai M, Searchfield G. Anxiety and depression, personality traits relevant to tinnitus: A scoping review. Vol. 55, International Journal of Audiology. Taylor and Francis Ltd; 2016. p. 605–15.

1. Will a chemical substance be administered?

yes  no

If yes:

a. Along what lines?

b. Name and origin of substance:

c. To whom is the reception, storage, distribution and return of unused chemical

substances?

d. Will radioisotopes be administered?

☐ yes ☐ no

Which?

1. If it is a new substance: has the investigator read the complete toxicological, animal, pharmacological and human dossier?  yes no

Not applicable

**14. Choice of subjects:**

a. Healthy people?

yes  no

Patients suffering from: Chronic tinnitus and/or chronic neck, head and/or jaw pain

b. Pregnant women or women who may become pregnant during the study?  yes  no

c. Number of subjects at UZ Gent: 212 (53 tinnitus patients with chronic pain, 53 tinnitus patients without chronic pain, 53 neck pain patients without tinnitus and 53 healthy controls)

d. Number of subjects external (in Belgium): <Click&TypeText>

**Note: the experiment is only insured for the number specified here.**

**If one wants to include additional participants, one will have to request this through an amendment.** e. Age: 18-65

f. Gender: male/female

g. How are they recruited? Via UZ Gent audiology department + via flyers at doctors, pharmacists, physiotherapists and social media

1. When is the participant expected to benefit?

a. Does the experiment have a diagnostic or therapeutic purpose that will bring immediate benefit to

the investigated person will bring?

yes  no

b. Is the experiment part of a diagnostic and therapeutic plan that can be expect the results to be useful to other sufferers in the foreseeable future?

yes  no

c. Is the experiment part of a set of studies whose diagnostic or therapeutic importance is not immediately apparent, but it may be expected that the results will later lead to diagnostic or therapeutic applications or to a better knowledge of physiopathological mechanisms?

yes  no

1. What interventions are specific to the study (beyond standard treatments), how frequently and for what length of time?

a. Purely clinical evaluations: clinical examination of the neck and jaw region (one-time)

b. Functional tests or dynamic tests (one-time):

- Audiogram and psychoacoustic measurements for tinnitus and hyperacusis (ENT/audiology service)
- Cognitive tests
- Listening effort test
- 3D kinematic analysis (Qualisys Motion Capture System)
- Shear Wave Elastography (SuperSonic Aixplorer MACH 30)
- Pressure pain thresholds (digital algometer)
- Heat detection thresholds (CHEPS device)
- Temporal summation (CHEPS device)
- Conditioned pain modulation (CHEPS device + hot water bath (Versacool))
- Electroencephalography (ANTneuro EEG machine)

c. Radiographic and/or isotopic investigations: NA

d. Blood sampling: NA

e. Tissue collection: NA

f. Others: Questionnaires (one-time):

- Tinnitus Functional Index
- Tinnitus Sample Case History Questionnaire
- Hyperacusis Questionnaire
- Central Sensitization Inventory
- Short Form Health Survey-36 items
- BIG 5 index 2
- Beck Depression Inventory
- Depression Anxiety and Stress Scale
- Pain Catastrophizing Scale
- Insomnia Severity Index
- Pittsburgh Sleep Quality Index

1. Taking into account the current data of science
   Do you believe that this study:

☒ probably poses no risk at all

☐ poses a possible risk.

What risk and frequency:

☐ very likely to present a risk.

Which risk and frequency:

b. What are the most common side effects of the preparation under study?

(side effects should also be clearly stated in the participant's information and consent form)

NA

1. Information and consent of subjects:

a. Willing adults

yes  no

Is subject consent obtained after a clear and objective explanation of the purpose of the study? Written:

yes  no

Oral:

yes  no

If no, why not?

In the latter case, is consent given by anyone other than the subjects?

yes  no

If yes, by whom?

Are there any special groups: in-house students, in-house staff?

These are not specifically recruited but by chance a student or staff member can participate in the study if that person meets the conditions for the study.

b. Incapacitated adults (= some psychiatric patients, persons unable to express their will, ...)

yes  no

Is consent given by anyone other than the subjects?

yes  no

If yes, by whom?

c. Children

yes  no

Is consent sought from their legal guardians?

yes  no

Is an information and consent form for children 12 and older provided?  yes  no

1. Is the subject information form attached?

yes  no

If no, why not?

1. Is the form for written consent attached?

yes  no

If no, why not?

1. During the course of this study, will individuals be under continuous medical supervision?

yes  no

a. Who is the supervising physician?

b. Will that supervision, if necessary, be assured during the hours following the study?

yes  no

c. If the person returns home during the hours following the examination, will in case of emergency

be able to quickly contact a physician?

yes  no

d. Name of this physician

1. Has insurance been taken out for the examination in accordance with the Belgian law of 7/5/2004? (The insurance certificate should be attached to the application if not insured by UZ Gent/UGent)

yes

By which insurance policy are you insured? (referring to an attached document is not sufficient)  UZ Gent

UGent

others + extent of coverage:

no, why not?

1. End date experiment

Date: woensdag 31 december 2025

**Please note that any experiment on people after the end date is no longer covered by the insurance so that**

**at that point you are in violation of the legal orders.**

**You can, however, extend the experiment subject to a new application.**

1. Financial agreement.
   If a final financial agreement is not yet available, a budget proposal counter-signed by a representative of the funder + researcher may suffice). If the amount of the final financial agreement exceeds the submitted budget proposal, that final financial agreement must still be submitted to the Medical Ethics Committee for approval.Financiële overeenkomst

not applicable

present with following subdivision:

honorarium

compensation for technical services
